# Light-induced photoreceptor and RPE degeneration involve zinc toxicity and are attenuated by pyruvate, nicotinamide, or cyclic light

**Published:** 2010-12-08

**Authors:** Christian T. Sheline, Yongdong Zhou, Shi Bai

**Affiliations:** Department of Ophthalmology and the Neuroscience Center of Excellence LSU Health Sciences Center, New Orleans, LA

## Abstract

**Purpose:**

Light-induced damage can be a problem after surgery or sun exposure. Short-duration, intense light causes preferential photoreceptor death in the superior central retina of albino mice and rats and serves as a model of oxidation-induced neurodegeneration. Previous work on retinal ischemia-induced neuronal death suggests the involvement of zinc (Zn^2+^) toxicity in the death and collapse of many retinal cell layers and demonstrates the protective efficacy of pyruvate. Retinal pigment epithelial (RPE) cells were shown to be sensitive to oxidative stress, and zinc, causing loss of nicotinamide adenine dinucleotide (NAD+) and adenine triphosphate (ATP), which was prevented by pyruvate and nicotinamide. We previously showed similar results in cortical neurons exposed to oxidative stress or Zn^2+^. In vivo, Zn^2+^ is normally present in the inner and outer segments (associated with rhodopsin), Bruch’s membrane and sclera (elastin), RPE, and the outer plexiform layer of the eye (synaptic). In this study, we examine the role of Zn^2+^ in oxidative stress and light-induced damage in vitro and in vivo.

**Methods:**

We modeled retinal toxicity in cell-culture lines derived from retinal tissue: Müller and human retinal pigment epithelial (ARPE-19) cells and a cone photoreceptor-derived line (661W). These cultures were exposed to Zn^2+^ and OS, and the therapeutic efficacy of pyruvate, nicotinamide, and NAD^+^ was determined. Sprague Dawley albino rats were exposed to 18 kLux of white fluorescent light for 1–4 h in the presence and absence of pyruvate, nicotinamide, lactate, and cyclic light. The intracellular free zinc concentration ([Zn^2+^]_i_) and cell damage were assessed 0.5 and 7 days later, respectively.

**Results:**

We show that Zn^2+^ and oxidative stress results in increased [Zn^2+^]_i_ and that Zn^2+^ therapeutic compounds (pyruvate, nicotinamide, and NAD^+^) and inhibitors of previously implicated pathways (sirtuin) are efficacious in vitro. Exposure to 18 kLux of cool white fluorescent light for 1 h induced a large increase in Zn^2+^ staining 4–14 h later, particularly in the superior outer nuclear layer and RPE of dark-maintained Sprague Dawley albino rats; 4 h of light was required to induce similar damage in cyclic light-maintained rats. Photoreceptors and RPE cells died in untreated animals at 3–7 days. However, nicotinamide and pyruvate (intraperitoneal), but not lactate, attenuated this death in treated animals, as measured using optical coherence tomography and confirmed by counting photoreceptor nuclei.

**Conclusions:**

Zn^2+^ plays a role in this injury, as suggested by the increased Zn^2+^ staining and the efficacy of Zn^2+^ therapeutics. These results suggest that cyclic light maintenance, Zn^2+^ chelation, pyruvate, and nicotinamide promote RPE and photoreceptor survival after injury and could be effective for various forms of retinal neurodegeneration. These results could have immediate clinical applications in surgery- or sun exposure- induced light damage to the retina.

## Introduction

There are many reasons to study light-induced damage to the retina: Light damage (LD) can occur under bright surgical lights if the eye patches normally used to block light are improperly placed or forgotten. Light damage also occurs during acute intense sun exposure (such as prolonged exposure over water or snow or improper solar eclipse viewing); chronic sun exposure has also been implicated in cataracts [1-5]. Furthermore, light-induced retinal damage is a physiologically relevant model for oxidation (OS)-induced retinal neurodegeneration [6,7]. Light or oxidation accelerates disease progression in many models of retinal diseases, such as retinitis pigmentosa (RP) mutant mice [8-11], Smith-Lemli- Opitz rats [12,13], and in retinal degenerations [14,15], including Royal College of Surgeons rats [16], age-related macular degeneration (AMD) [17], glaucoma [18], Tubby mice [19], and diabetic retinopathy [20]. This was reviewed in [21]. Thus, therapeutics that are efficacious against oxidation- or light-induced damage should also be effective against these retinal degenerative diseases.

Retinal ischemia-mediated neuronal death is associated with an increase in zinc (Zn^2+^), and pyruvate or nicotinamide attenuates this death in vitro and in vivo by restoring NAD^+^ levels [22,23]. Intra-ocular injection of 1–10 nanomoles of the Zn^2+^ chelators, EDTA-calcium salt (CaEDTA), or N,N,N'N'-tetrakis(-)[2-pyridylmethyl]-ethylenediamine (TPEN) attenuates neuronal death induced by retinal ischemia [24]. Higher levels of these chelators can induce retinal detachment [22]. Either extracellular Zn^2+^ uptake through voltage gated Ca^2+^ channels or OS-induced intracellular Zn^2+^ release from Zn^2+^ binding proteins or organelles are neurotoxic. Previous chelator, metabolic, and zinc transporter 3 (ZnT3 and slc30a3) knockout studies have demonstrated that synaptically released zinc contributes to neuronal death after global ischemia, hypoglycemia, or seizures [25-27]. Intracellular Zn^2+^ release contributes to focal ischemia, OS-, or trophic deprivation-induced neuronal death [28,29]. Pyruvate or nicotinamide can attenuate these zinc neurotoxicities in vitro and in vivo in part through restoration of NAD^+^ levels lost due to Zn^2+^ exposure; this NAD^+^ restoration restores glycolytic flux [28,30-33]. We are interested in the role of excess zinc in retinal degeneration as a result of pathologic light exposure and oxidative conditions. In ambient light, Zn^2+^ is present physiologically in layers of the eye, most notably in the rod inner and outer segments (RIS, ROS) of the outer nuclear layer (ONL), the outer plexiform layer (OPL), and retinal pigment epithelial (RPE) cells. However, in the dark, the RIS and ROS segmental Zn^2+^ disappears and Zn^2+^ appears in photoreceptor perikarya of the ONL. Zn^2+^ serves important functions, such as modulating neurotransmission [34,35], regulating intracellular metabolism, and dark-light adaptation in retina [reviewed in 36]. The “stainable, releasable” Zn^2+^ has been demonstrated through auto-metallographic staining [36-38] and fluorescent dye staining [22,36,39] and Zn^2+^ has been shown to be synaptically released in the OPL through photoreceptor activity [39,40].

The pathway we studied was that elevation of intracellular Zn^2+^ can cause a reduction in NAD^+^ and ATP levels, causing dysfunction of glycolysis and cellular metabolism [30,32]. In the experiments presented here, we used the light-induced retinal damage model in albino rats. Oxidative stress has been shown to be involved in this model in the initiation of damage after intense light exposure. In addition, endoplasmic reticulum (ER) stress has also been shown to occur after LD and in rhodopsin RP mutants, and degeneration could be attenuated by overexpression of ER chaperone proteins, perhaps reflecting excessive shedding or misfolding of ROS proteins [41,42]. Previous studies have shown that intense light-induced photoreceptor and RPE cell death is greater in the superior central retina compared to the inferior retina, as measured by ONL thickness and counting [43,44]. This damage involves specific prolonged apoptotic rod cell death and rapid necrosis cone cell death; the roles for DNA damage and repair have been established [reviewed in 21,43]. In our study, we investigated whether light- and radical-induced damage of retinal cells or cell lines causes toxic zinc accumulation, and whether pharmacologic Zn^2+^ therapeutics, which restore NAD^+^ levels, are effective in vitro and in vivo.

## Methods

### Cell culture

A Müller cell line and the ARPE-19 cell line were maintained in Dulbecco’s Modified Eagle Medium (DMEM) media containing 10% fetal bovine serum and penicillin/streptomycin. The 661W cone photoreceptor cell line was maintained in the same medium with supplementation of 2-mercaptoethanol, hydrocortisone 21-hemisuccinate, progesterone, and putrescine [45-47]. Some 661W cultures were preloaded and grown with an additional 10 μM Zn^2+^ for 2 days before toxicities (~13 μM Zn^2+^ total in plating medium). This Zn^2+^ concentration does not cause toxicity or affect growth, but does increase basal [Zn^2+^]_i_ [28]. Cells were grown in 5% CO2 in 95% humidity at 37 °C for 2 days after splitting to achieve 50%–70% confluency at the time of the experiment. Cells were exposed to zinc (10–200 μM), ethacrynic acid (ETH; 15–100 μM), and H_2_O_2_ (100–400 μM) for 24 h in minimal essential medium.

### Cell viability assay

The cells (50%–70% confluent) were exposed to zinc, ETH, and H_2_O_2_ in the presence of 1–10 mM pyruvate, NAD^+^, or nicotinamide, and 0.03–0.5 μM TPEN or 3–20 μM of the sirtuin pathway inhibitor, sirtinol. Cell viability was determined by measuring lactate dehydrogenase released into the medium after 24 h. For cells that were exposed to NAD^+^, cell viability was determined by MTT assay or PI staining assay [48]. Twenty-four h after exposure, cells were stained with 0.1% MTT for 30 min at 37 °C, lysed, and absorbance measured at 595 nm, or PI was added (5 μg/ml) for 30 min at 37 °C and fluorescence measured (ex 530/em 645).

### Live-cell imaging

FluoZin3 AM (5 μM; Invitrogen/Life Technologies, Carlsbad, CA) was pre- or post-loaded in the cell lines for 30 min at 37 °C, washed, and exposed to toxic Zn^2+^, ETH, or H_2_O_2_ for 1–11 h before photomicrographs of identical duration were taken.

### Rat light damage model

Sprague Dawley albino rats (Charles River, Wilmington, MA) weighing 150–175 g were acclimated for 5 days to a cyclic, dim overhead fluorescent light (30 Lux), followed by a 60 h dark adaption. At this point, one group of animals (dark) was maintained totally in the dark for the duration of the experiment, using red light illumination to dilate their eyes (1% tropicamide ophthalmic solution USP), and was returned to their cages after 1–2 h of light exposure. The second group of rats (light) had their eyes dilated in room light after the 60 h dark adaptation, were exposed to 4 h of light damage, and were returned to cyclic light after 24 h recovery in the dark. The animal chamber was rotated during the light exposure to ensure that the animals were awake with their eyes open. Rats were exposed to bright cool white fluorescent light from 8x 20W-circular fluorescent bulbs (18,000 Lux) [43] in the presence or absence of 500 mg/kg intraperitoneal (i.p.) pyruvate or nicotinamide (3×/week). This was followed by recovery in the dark for 24 h (light) or 7 days for the dark maintained animals (dark). After 24 h recovery in the dark, the (light) group was returned to the cyclic, dim overhead fluorescent light environment for 6 days. All studies were conducted within the guidelines (stated above) established by the Institutional Animal Care and Use Committee (Louisiana State University Health Sciences Center, New Orleans), and were in accordance with the PHS Guide for the Care and Use of Laboratory Animals, USDA Regulations, and the AVMA Panel on Euthanasia guidelines.

### Optical coherence tomography

Optical coherence tomography (OCT) is an optical signal acquisition and processing method providing extremely high-quality, micrometer-resolution, three-dimensional images from within optical scattering media (Spectralis, Heidelberg Engineering, Heidelberg, Germany). On the 7th day after light damage, rats were anesthetized with ketamine and xylazine and OCT was performed to measure the thickness of the ONL. A real-time eye tracker was used to couple cSLO and SD-OCT scanners to position and stabilize the OCT scan on the retina. Scaling X was 3.24–3.31 µm/pixel; scaling Z was 3.87 µm/pixel. The built-in scale bar was used when performing OCT analysis. The thickness of the ONL was measured from the bottom edge of the outer plexiform layer to the top edge of the RIS.

### Retinal histology

After OCT, rats were sacrificed by CO_2_ asphyxiation. Eyes were fixed in 2% formaldehyde/2% glutaraldehyde and cut in half along a superior-to-inferior meridian through the center of the optic nerve. After a 1 h fixation period in 1% osmium tetroxide and sequential dehydration in ethanol, eyes were embedded in plastic resin (Electron Microscopy Systems, Hatfield, PA). Retinal sections of 1.5 microns were cut, mounted on glass slides, and stained with 0.1% toluidine blue. The number of ONL nuclei was counted on sections from 6 different retinas at increasing distances from the optic nerve on the superior-to-inferior meridian. Pictures were taken in the mid-superior and mid-inferior hemispheres.

### Retinal Zn^2+^ staining

Eyes of Sprague Dawley rats were collected 4 and 14 h after 1 or 4 h of light exposure with or without dark maintenance, respectively. For example, 4 h LD (light) + 4 h refers to an animal that had its eyes dilated in normal light (not red light) before undergoing 4 h of light damage; the animal was then sacrificed 4 h after light damage. Fresh frozen cryostat sections (10 microns) were prepared, dried, and stained with 5 μM ZinPyr-1 (ZP1, TefLabs, Galveston, TX) for 2 min, washed with PBS, and then photomicrographs were taken immediately using the exposure times indicated (ex: 480nm; em: 530nm). Note that to prevent complete saturation of the images, exposure times were varied. There was no autofluorescence at this wavelength either basally or after light damage, and staining was prevented by pretreatment with the zinc specific chelator, TPEN (data not shown).

### Reagents

All materials were purchased from Sigma Chemical Co. (St. Louis, MO) unless otherwise stated.

## Results

### Zinc and oxidative stress induce [Zn^2+^]_i_ increases in cone photoreceptors, ARPE-19 cells, or Müller cells

**Figure 1:**
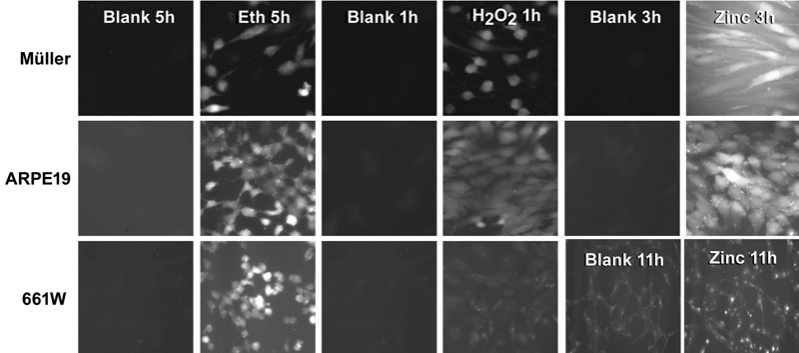
Zinc (Zn^2+^), or oxidative stress induces an increase in intracellular free zinc concentration ([Zn^2+^]_i_). Cultures of Müller, ARPE-19, and 661W cells were pre- or post-loaded with 5 μM FluoZin3 AM and exposed to 60–300 μM Zn^2+^, 30–60 μM ethacrynic acid, and 200–400 μM H_2_O_2_, as indicated. Representative photomicrographs (n=6) of identical exposure were taken at the times shown.

We examined whether zinc (30–200 μM), ETH (10–100 μM), and H_2_O_2_ (100–400 μM) induced a [Zn^2+^]_i_ increase in Müller, ARPE-19, or 661W cone photoreceptors. Cultures were pre- or post-loaded with 5 μM FluoZin3 AM for 30 min and then washed out and exposed to oxidative stress as indicated. We found that after 1–11 h, [Zn^2+^]_i_ was increased after Zn^2+^ or oxidative exposure in each of these cell lines (Figure 1). The increase in Fluo-Zin3 staining was specific for Zn^2+^ as demonstrated using the intracellular zinc chelator, TPEN. The extracellular zinc chelator, CaEDTA, was ineffective except for exogenous Zn^2+^ exposure, perhaps due to the intracellular release of zinc and the short 1–5 h exposure to CaEDTA (data not shown). Pyruvate, nicotinamide, or NAD^+^ are not expected to affect [Zn^2+^]_i_ because they had no effect on [Zn^2+^]_i_ in cortical neurons, but did restore NAD^+^, glycolytic flux, and prevented Zn^2+^ neurotoxicity.

### Zinc- and oxidative-stress-induced death of cone photoreceptors or Müller cells was attenuated by Zn^2+^ therapeutics

**Figure 2:**
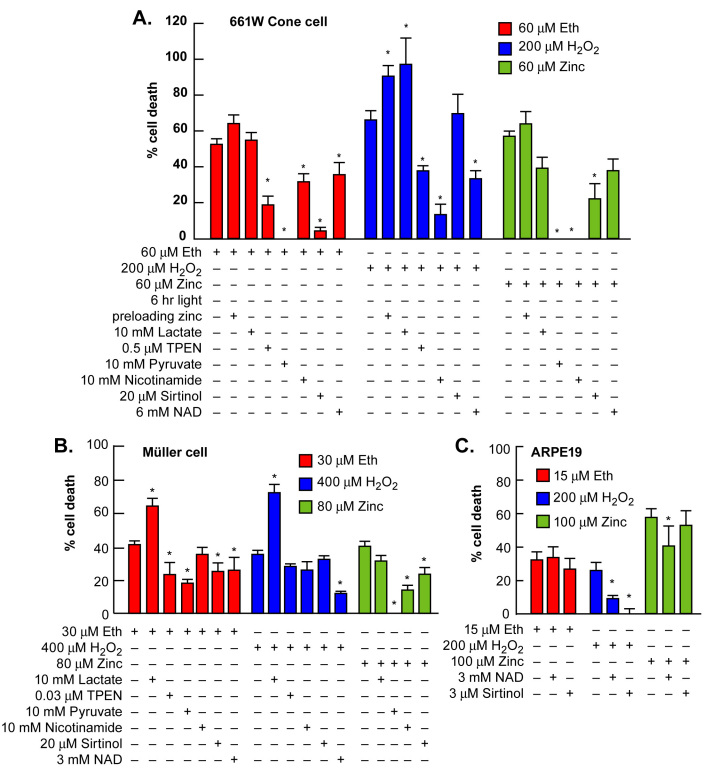
Zn^2+^ or oxidative-stress-induced death was attenuated by Zn^2+^ therapeutics. **A**: Pyruvate, nicotinamide, NAD^+^, TPEN, and sirtinol variably attenuated ethacrynic acid, H_2_O_2_, or zinc toxicity in the 661 W cone photoreceptor cell line. **B**: The effect of these exposures on Müller glial cells is presented. **C**: The effect of these exposures in ARPE-19 cells is presented (n=10–15). * indicates difference from toxic exposure alone at p<0.05 by one-way ANOVA and a Bonferroni test.

TPEN, pyruvate, nicotinamide, NAD^+^, or sirtinol (Zn^2+^ therapeutics) can protect cortical neurons from zinc and oxidative toxicities in vitro or in vivo [28,30,32]. Chronic addition of these Zn^2+^ therapeutic compounds attenuated zinc and oxidative-stress-induced death of Müller or ARPE-19 cells and 661W cone photoreceptors as measured using lactate dehydrogenase release to the media, 5 μg/ml PI staining, or 0.1% MTT staining [49]. Zn^2+^ preloading potentiated oxidative-stress-induced toxicity in 661W cells. The respective LD_50_ for zinc, ethacrynic acid, and H_2_O_2_ was 60 μM, 60 μM, and 200 μM for 661 W cells; 100 μM, 30 μM, and 600 μM for Müller cells; and 90 μM, 20 μM, and 300 μM for ARPE-19 cells. Chronic additions of 3–10 mM pyruvate or nicotinamide or 3–6 mM NAD^+^ were consistently efficacious across toxicities. The resultant cell death was variably attenuated by 3–20 μM sirtinol, whereas 10 mM lactate was consistently ineffective or detrimental. Zn^2+^ pre-loading potentiated, and 0.03–0.5 μM TPEN attenuated these injuries (Figure 2). The effective concentration of TPEN was lower in Müller cells due to their tendency to detach from the plate at higher concentrations of TPEN.

### Light damage increases Zn^2+^ staining in photoreceptors

**Figure 3:**
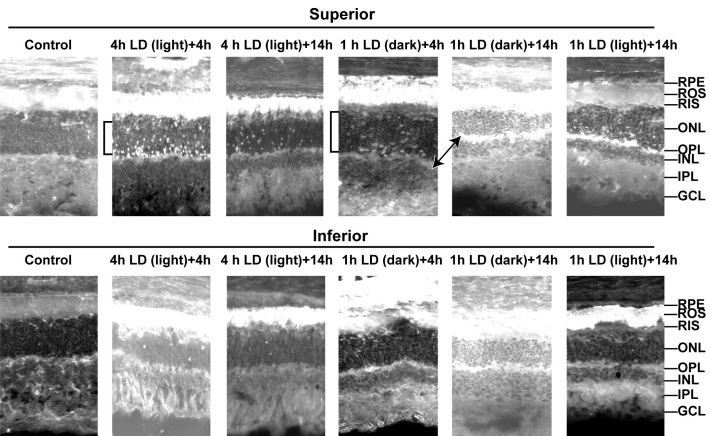
Light-induced damage caused Zn^2+^ accumulation preferentially in superior photoreceptors, retinal pigment epithelial (RPE), rod outer segments (ROS), and outer plexiform layer (OPL). Light-induced damage (LD) was performed and fresh frozen rat retinas were analyzed at 4 or 14 h after 1 h of light damage in dark maintained conditions (dark) or after 4 h of light damage in non-dark maintained conditions (light). Zn^2+^ accumulation (white regions) was assessed by ZP1 staining of fresh frozen rat eye cryostat sections cut at 10 microns, which were dried and stained in 5 μM ZP1 for 2 min. Representative photomicrographs (n=4) were taken of the mid inferior and mid superior regions of the retina at 5 s exposure for: control, 4 h LD (light) + 4 h, and 1 h LD (dark) + 4 h; at 1 s exposure for: 1 h LD (dark) + 14 h; and at 2 s exposure for: 4 h LD (light) + 14 h, and 1 h LD (light) + 14 h. Layers are as marked. Notice the large increase in the number and intensity of Zn^2+^ stained cells in superior ONL (brackets), ROS, and RPE. Light=non-dark maintained, Dark=dark maintained.

We performed time courses of zinc accumulation after light-induced damage using a zinc fluorescent dye (5 μM ZP1) on fresh frozen dried rat retinas. Figure 3 shows that zinc accumulated preferentially in the mid-superior RPE and the superior photoreceptors of the ONL at 4–14 h after light damage, which is before the onset of cell death induced by light (48–96 h). The two previously mentioned layers are the most sensitive to LD. Zinc staining started at 2 h, and was maintained at 24 h after LD (data not shown). More zinc-stained photoreceptors were present in the mid-superior than in the mid-inferior retina, correlating with the sensitivity of these regions to LD. The stain was specific for Zn^2+^ because at 480 nm excitation, there was no autofluorescence and no staining after TPEN pretreatment (data not shown). We also show that Zn^2+^ staining and accumulation in ROS, RIS, and photoreceptors after only 1 h of LD was much more prevalent when animals were kept dark-adapted and if the eyes were collected and frozen in the dark (note exposure times). This may be due to the depolarization of photoreceptors in the dark, which allows Zn^2+^ influx through voltage-gated Ca^2+^ channels [50]. Because Zn^2+^ staining must be performed on fresh frozen dried tissue sections, the morphologic preservation of the tissue was not perfect. However, the inner and outer segments, ONL, RPE, and plexiform layers were still easily discernible, and the increase in the number of superior photoreceptors and RPE cells that stained for Zn^2+^ at 4–14 h was greater than the increase in inferior photoreceptors and RPE cells. The entire superior ONL, ROS, RIS, and RPE layers were destroyed after 3–7 days (see below), and these were the layers that preferentially stain with Zn^2+^ 4–14 h after LD.

### Light damage induced death of photoreceptors in vivo

**Figure 4:**
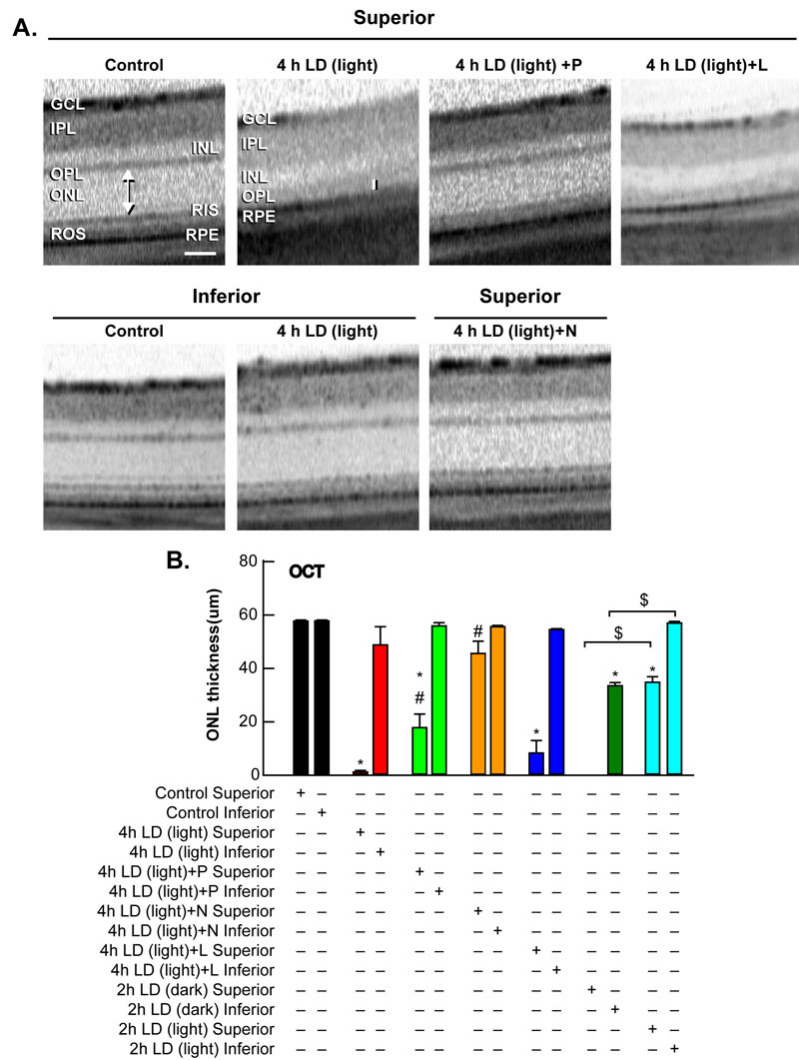
Optical coherence tomography (OCT) scans demonstrate protection of ONL by pyruvate and nicotinamide against LD. **A**: Representative ONL images were taken from OCT in control, 7 days after 4 h light damage in non-dark conditions (4 h LD (light)), 4 h LD (light) + P, 4 h LD (light) + N, and 4 h LD (light) + L, where P, N, and L refer to 500 mg/kg i.p. injection before LD and 3x/week of pyruvate, nicotinamide, or lactate, respectively. Bar represents 50 microns. **B**: The mean thickness in central superior and central inferior hemispheres (arrow or bar in **A**) of the retina in microns after these exposures is presented ± SEM (n=6). * indicates difference from control, # indicates difference from light damage, and $ indicates difference between dark maintained and non-dark maintained retinas at p<0.05 by one-way ANOVA and a Bonferroni test.

**Figure 5:**
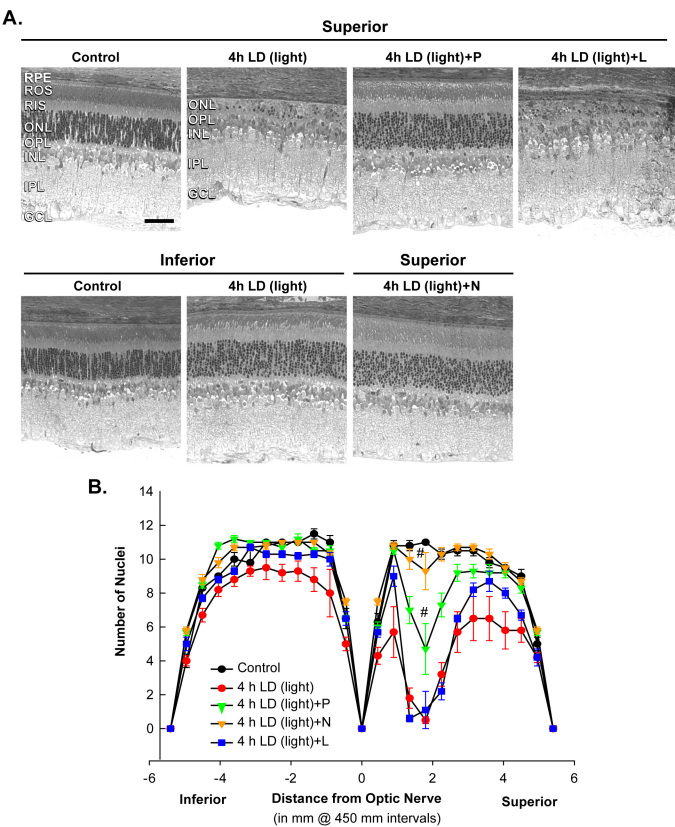
Plastic sections confirm protection of ONL by pyruvate and nicotinamide against LD. **A**: Plastic sections were cut from eyes along a superior to inferior meridian encompassing the optic nerve and stained with 0.1% Toluidine Blue. Representative photo-micrographs were taken of the mid superior and mid inferior regions from each of the groups as designated in Figure 4. Bar represents 50 microns. **B**: The mean number of nuclei at increasing distance from the optic nerve on the superior and inferior sides was averaged and plotted as a function of distance from the optic nerve for each of the experimental conditions above (n=6). # signifies difference from LD at p<0.05 by one-way ANOVA and a Bonferroni test.

**Figure 6:**
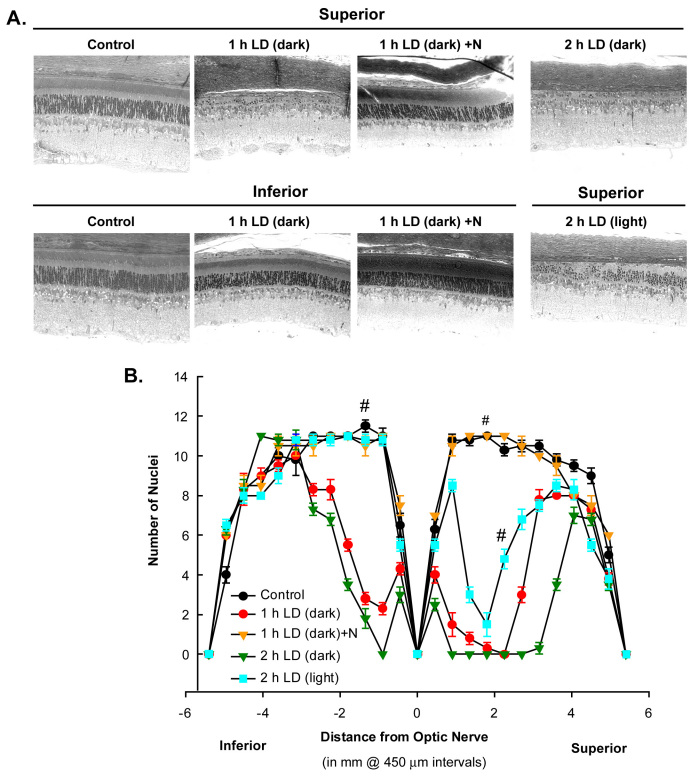
Plastic sections confirm potentiation of ONL loss by dark maintenance and protection by nicotinamide against LD. **A**: Plastic sections were cut from eyes along a superior to inferior meridian encompassing the optic nerve, and were stained with 0.1% Toluidine Blue. Representative photo-micrographs were taken of the mid superior and mid inferior regions from Control group; 1 h light-induced damage dark-maintained (1 h LD (dark)) group; LD + i.p. injection of nicotinamide (1h LD (dark) + N); and 2 h of LD dark maintained (2 h LD (dark)) versus non dark maintained (2 h LD (light)) as marked. **B**: The mean number of nuclei at increasing distance from the optic nerve on the superior and inferior sides was averaged and plotted as a function of distance from the optic nerve for each of the experimental conditions above (n=6). # signifies difference from LD at p<0.05 by one-way ANOVA and a Bonferroni test.

Treatment with pyruvate or nicotinamide (i.p.) attenuated damage to the superior retina, as shown 7 days post-LD by optical coherence tomography (Figure 4), and by ONL cell counting of plastic sections (Figure 5 and Figure 6). Rats exposed to 4 h of 18 kLux white fluorescent light followed by cyclic light displayed complete loss of the superior central ONL, including the RIS, ROS, and the RPE, and ~10% photoreceptor death in the inferior central ONL, as previously reported [43,44,51]. Pre-treatment with i.p. pyruvate, but not lactate, afforded substantial protection of the photoreceptors and RPE cells against light damage, and partial protection of RIS and ROS (Figure 4 and Figure 5); nicotinamide provided complete protection to photoreceptors, RIS, ROS, and RPE cells (Figure 4, Figure 5, and Figure 6). Quantitations of ONL thickness, measured as the distance between the RIS and the OPL (arrow or bar), were made by OCT, and are plotted in Figure 4B. Figure 5B presents the number of photoreceptor nuclei at increasing distances superior or inferior to the optic nerve.

### Data analysis and statistics

The changes in retinal cell death were determined in cultures under the conditions stated. Means±SEM are plotted and the number is given for each experiment in the figure legends. Results were compared to sham wash or saline injection controls and toxin or injury exposure alone. A one-way ANOVA was used to assess variance in each set of experiments, followed by a Bonferroni test. Significance was achieved by a p value of less than 0.05.

## Discussion

In these experiments, we show that: 1) Intense light can induce zinc accumulation in the retina, particularly in the RPE, ROS, RIS, sclera, OPL, and photoreceptors. The early, preferential accumulation of zinc in the superior photoreceptors after light-induced damage (before ONL cell death) suggests that zinc may play a role in the death of these cells in vivo. Potentiation of Zn^2+^ accumulation and injury by maintenance of dark adaptation/depolarization supports this notion. 2) The superior retina is more sensitive to light-induced damage compared to the inferior retina, and pyruvate or nicotinamide (i.p.) can attenuate photoreceptor cell death as shown by OCT and plastic sections. 3) In vitro, pyruvate, nicotinamide, sirtinol, NAD^+^, and, perhaps, TPEN can attenuate Müller, ARPE-19, and 661W cone cell death induced by zinc and oxidative stress.

We and others have previously shown that Zn^2+^ accumulates in cortical, thalamic, and striatal neurons exposed to extracellular Zn^2+^ or oxidative stress, and that Zn^2+^ chelators or compounds that restore NAD^+^ levels and glycolysis (Zn^2+^ therapeutics) attenuate injury [28,30,32,52,53]. We now show that these Zn^2+^ therapeutics were also effective in retinal cells in vitro and in vivo. These compounds have been shown to prevent Zn^2+^ neurotoxicity by restoring NAD^+^ levels and glycolytic flux; this may be their mechanism of action against light-induced damage as well. The mechanisms of Zn^2+^ toxicity has been shown to be multi-factorial, and they include affects on increasing autophagy [54], damaging mitochondria inducing metabolic dysfunction [55,56], inhibiting protein phosphatases causing kinase cascade activation [53,57], and inducing OS through activation of nicotinamide adenine dinucleotide phosphate oxidase [58-60]. Importantly, chelator compounds, Zn^2+^ reducing manipulations, and manipulations demonstrated to restore NAD^+^ (pyruvate, nicotinamide, poly-ADP ribose polymerase (PARP) inhibition, slow Wallerian degeneration (Wld^s^)) have displayed in vivo efficacy against Zn^2+^ neurotoxicity, but the alternate pathways described above have limited evidence for in vivo efficacy [22,28,31-33,61-64].

High levels of Zn^2+^ also have been found in the aggregated proteins that comprise drusen in AMD [65]. This is similar to the presence of high levels of Zn^2+^ in amyloid plaques of Alzheimer disease [66,67]. Zn^2+^ chelation has been proposed as a therapy for Alzheimer disease [68], and a chelator from DPharm (DP-b99) is being tested in phases II and III Alzheimer clinical trials. This chelator also has proven effective against stroke [69]. This is in contrast to the use of Zn^2+^ as a supplement in the AREDS trial against AMD. Zn^2+^ was used in the AREDS trial because it has been reported to be beneficial in the immune system, where it may cause an antioxidant response [reviewed in 70]. However, due to Zn^2+^ accumulation after ischemia, injury, and in drusen, many researchers have recently questioned the use of Zn^2+^ supplementation in AMD treatment.

However, it should also be noted that too little Zn^2+^ in the retina has been shown to be detrimental. Zn^2+^ deficiency in patients with chronic alcoholic cirrhosis or pancreatitis may reversibly impair dark adaptation, electroretinography, and induce structural defects in the periphery of the retina [71,72]. Also, a chronic Zn^2+^ deficient diet [73], or Zn^2+^ chelation [74], resulted in osmiophilic lipid inclusion bodies in the RPE, and ROS disruption.

In these experiments on the role of Zn^2+^ in retinal neurodegeneration, we used a light-induced retinal damage model for albino rats. Light damage has been shown to cause both direct DNA damage and oxidative DNA damage, both of which can activate PARP, resulting in NAD^+^ loss [75]. Furthermore, light damage in the eye has been shown to activate PARP, and antioxidants attenuate PARP activation and LD of photoreceptors [76]. We performed time courses of zinc accumulation after light-induced damage using a zinc fluorescent dye on fresh frozen rat retina to demonstrate the accumulation of zinc in the RPE and ONL before light-induced cell death. This Zn^2+^ accumulation was especially prevalent 4–14 h post-exposure in the superior versus the inferior central retina, which is where photoreceptors are most vulnerable to light damage. Zn^2+^ accumulation peaked more than 20 h before photoreceptor death occurred, and it was most prevalent in the ROS and in dark-maintained animals. RPE cells also accumulated significant Zn^2+^, which may be the result of their involvement in the clearance of shed outer segments that contain significant Zn^2+^ in association with rhodopsin. Zn^2+^ has been shown to be a structural component of rhodopsin, which is critical for its stability, cis-retinal binding, and function in the dark state [77,78]. In addition, substantial Zn^2+^ staining also occurred in the sclera, especially after light damage. This was likely due to the tight binding of zinc by the membrane protein elastin.

Photons are absorbed in the outer segments by rhodopsin, changing its structure and resulting in activation of the phototransduction cascade and photoreceptor hyperpolarization. Therefore, light stimulation induces hyperpolarization and dark adaptation induces photoreceptor depolarization, which potentiates Zn^2+^ influx through voltage-gated calcium channels [50,79,80]. A similar situation seems to occur in the retina, where LD and zinc accumulation was greatest if the retina was completely dark adapted (and therefore depolarized) before and after LD [81,82]. These data implicate Zn^2+^ influx into photoreceptors in LD. Also, the stainable Zn^2+^ pool in the retina is dramatically affected by dark adaptation. As stated previously, Zn^2+^ is located in specific retinal layers in adults exposed to ambient light, but Zn^2+^ is especially found in the inner segments of photoreceptors. Dark adaptation results in the stainable zinc pool disappearing from the RIS and appearing in the cell bodies of most photoreceptors [36]. The light-dark staining pattern in RIS can be explained by an interaction with rhodopsin (see below), but the pattern in photoreceptor perikarya suggests an unknown Zn^2+^ binding/translocation protein interaction. It is also interesting to note that Zn^2+^ deficiency results in a loss of night vision [71,83], which suggests that this dark-induced change in Zn^2+^ staining may be functionally relevant.

Grimm and colleagues demonstrated effective LD using only 2 h of exposure to 15 kLux white light when pigmented mice were completely dark-maintained before and after LD [84]. Mice maintained in this manner are more sensitive to LD, perhaps due to depolarization of photoreceptors in the dark state and the movement or release of Zn^2+^ from the inner segments to the photoreceptor cell bodies, which may potentiate injury (Figure 3B) [36]. Some researchers have not demonstrated this degeneration in pigmented mice with 2 h LD, but the dark-adapted state may not have been maintained. In Figure 4B and Figure 6, we show that 2 h of LD with dark maintenance causes substantially more injury than if the albino rats are not dark-maintained. Only 1 h of LD causes almost complete loss of superior photoreceptors in albino Sprague Dawley rats (as determined by OCT and plastic sectioning), while 4 h of light damage was required if animals were returned to light.

Where does the Zn^2+^ come from to induce the massive increase in Zn^2+^ staining observed in the ROS and ONL after LD? In the inner and outer segments, rhodopsin has been shown to bind to and require one ion of Zn^2+^, which stabilizes the structure and organization of rhodopsin into disc membranes [85,86]. The amino acids involved in this high-affinity, required, tetrahedral Zn^2+^ binding site are Glu^122^ (at the end of transmembrane region 3), and His^211^ (at the end of transmembrane region 5). This Zn^2+^ coordination site lies within the 11-cis-retinal binding pocket and is critical for the stability of this chromophore-receptor interaction and for proper rhodopsin folding in the inactive (dark) state. Binding of additional Zn^2+^ ions (> 1) to rhodopsin through lower affinity sites (His^100^ and His^195^) has been suggested to induce a destabilization that might help RPE cells (with a high basal Zn^2+^ content) degrade rhodopsin from shed outer segments. Many of the RP rhodopsin mutations cluster around the amino acids involved in Zn^2+^ binding (His^100^ and His^195^), and the His^211^-Pro or -Arg and the Pro^23^-His RP mutations have been suggested to affect Zn^2+^ binding and reduce rhodopsin stability [77,78]. Light activation also induces structural changes increasing the distance between TM 3 and 5 of rhodopsin, which increases the tetrahedral binding distance for Zn^2+^; this is inconsistent with continued Zn^2+^ binding [77,87]. We therefore predict Zn^2+^ release from rhodopsin by light exposure, which is supported by our data (Figure 3). Rhodopsin (Rho) knockout (KO) mice lack opsin synthesis, do not develop an ROS structure, and do not activate the required apoptotic AP-1/c-fos transcription factor in response to LD. Photoreceptors from Rho KO mice spontaneously degenerate starting at 3 months of age, but at 1–2 months of age Rho KO photoreceptors are resistant to LD [84,88,89]. RPE65 KO prevents re-isomerization of all-trans retinol in the visual cycle, and thereby prevents rhodopsin regeneration and rod function. RPE65 KO mice also do not show any photoreceptor death after LD [84,90]. C-fos/AP-1 KO mice were also insensitive to LD [91] and increased [Zn^2+^]_i_ activates kinase cascades that activate c-fos/AP-1 [53,58,92]. A diffusible light damage-induced factor has been postulated in the literature [21,36,84], and it is possible that Zn^2+^ is this factor. We postulate that Zn^2+^ is released from rhodopsin by light and moves to the RIS and cell body, allowing Zn^2+^ staining and c-fos/AP-1 activation. The regenerated rhodopsin then rebinds this zinc in the ER of the RIS in the dark and RIS staining goes away, thus accounting for the RIS light-dark Zn^2+^ staining pattern.

Immediate post-LD treatment with these compounds is ongoing and may be effective since it has been shown that pyruvate and nicotinamide are effective for 1–2 h after ischemia, hypoglycemia, and trophic deprivation [26,28,30-33]. Studies involving eye drop application of pyruvate or nicotinamide are also ongoing, and this route of administration may be effective because topical application of pyruvate was shown to penetrate the human cornea [93]. We are also studying the efficacy of reducing retinal Zn^2+^ through chelators and diet to affect Zn^2+^ staining and LD, as well as the potentiation of LD by dark maintenance. Finally, we plan to examine the zinc staining of the Rho and RPE65 KO animals and the efficacy of pyruvate and nicotinamide against rhodopsin RP mutant models of retinal degeneration. These experiments have validated the therapeutic efficacy of pyruvate and nicotinamide against the light-induced retinal damage model in rat and have implicated an increase in retinal Zn^2+^ accumulation in this injury.
